# Items

**Published:** 1898-01

**Authors:** 


### Items.

Fined Under the Medical Registration Law.—In the Municipal Court at Boston, recently, Judge Ely fined Augustus R. Gilmore $200 for styling himself a "doctor" on his cards. The defendant claims to effect cures by the laying on of hands. He appealed and was held in $500 bail for the upper court.—*Boston Medical and Surgical Journal, December 16, 1897.*

A certain eastern firm has been doing some very unique and attractive advertising of friable pills. We recently had some experience with the pills, however, which makes us a little cautious about believing all that advertisers may say. Commencing in the morning, we gave a little fellow during the day eight one-grain quinine pills of the friable variety. No result followed, but the nurse next morning showed six of these which had passed intact through the alimentary canal. The trouble was, the poor little mortal did not carry in his stomach a hammer and board with which to crack them open. A pill to do good must be covered, if covered at all, with something that the juices of the digestive tract will act upon, and not necessarily that can be crushed between the thumb and finger. The average human being does not carry a hammer in his stomach.—*Western Medical Review.*

The Medical Society of the State of New York will hold its ninety-second annual meeting at the Germain Hall, Albany, Tuesday, Wednesday and Thursday, January 25, 26 and 27, 1898, under the presidency of Dr. Seneca D. Powell, of New York. Business committee: Dr. W. G. Macdonald, chairman, 27 Eagle street, Albany; Dr. Ernest Wende, 471 Delaware avenue, Buffalo, and Dr. B. Farquhar Curtis, 7 E. 41st street, New York.

The American Pediatric Society, in making a collective investigation of infantile scurvy in North America, sends out the following circular:

The Committee for the Collective Investigation of Infantile Scurvy, appointed by the American Pediatric Society at its meeting in 1897, requests reports upon cases of this disease, whether previously published or otherwise. Will you kindly answer the questions appended as fully and as promptly as possible ? Please write out the answers ; do not cross out the questions. Answers may be sent to, and additional blanks obtained from, any of the members of the committee. Full credit will be given, and no case will be used in such a way as to interfere with its subsequent publication by the observer. A final printed report of the investigation will be sent to those furnishing cases. When the blank space allotted is not sufficient for a complete answer, please write the answer upon the back of the sheet, numbering and lettering it properly, and writing "over" opposite the printed question. Will you kindly notify us of other physicians whom you believe to have had cases of infantile scurvy under their care.

Returns close April 1, 1898.

[Signed].

J. P. Crozer Griffith, M. D., Chairman, 123 S. 18th St., Phila.
William D. Booker, M. D., 853 Park Ave., Baltimore.
Charles G. Jennings, M. D., 457 Jefferson Ave., Detroit.
Augustus Caille, M. D., 753 Madison Ave., New York City.
John Lovett Morse, M. D., 317 Marlboro St., Boston.

Committee.

L. EMMETT HOLT, M. D., President.

Requests for blank forms may be sent to Dr. Irving M. Snow, 476 Franklin street, Buffalo, N. Y.

---

Mid-Winter Sun Baths.—The Clyde line is advertising mid-winter sun baths in connection with its Florida service. This is not a bad idea, as it is a well-known fact that the sun shines in Florida almost every day in the year. Truly, this tropical state is a "land of flowers and sunshine" and nature in all her glory reigns supreme there every day in the year. Thither the ailing ones have gone by the thousands ; they return only to tell the delightful tales of this big and hospitable state. First, the old folks went to Florida, but now the old and young both congregate at the various Floridian resorts to do homage to nature, by nature blest and benefited in health and happiness. The Clyde line of steamers from New York to Jacksonville is the ideal way to reach the sunny South. The steamers from New York to Charleston make the trip in about two days ; at the latter point they stop just long enough to give the passengers an opportunity to see this old and historic city. Thence the magnificent steel steamers wend their way to Florida, landing in Jacksonville in daylight, making the trip in all about three days from New York to the metropolis of the most tropical state in America.

